# Current Overview and Main Challenges of the Use of *Galleria mellonella* in Biomedical Research

**DOI:** 10.1002/cpz1.70361

**Published:** 2026-04-16

**Authors:** Fabiana Alves de Souza Silva, Maria Eduarda da Silva Costa, Patrícia Michelle Nagai de Lima, Jaqueline Lemes Ribeiro, Betina Sayeg Burd, Rondinelli Donizetti Herculano, Laura Beatriz Borim da Silva, Paulo Henrique Fonseca Carmo, Luis Augusto de Almeida‐Silva, Kevin Kavanagh, Juliana Campos Junqueira, Maíra Terra Garcia

**Affiliations:** ^1^ Department of Biosciences and Oral Diagnosis, Institute of Science and Technology Universidade Estadual Paulista (UNESP) São José dos Campos Brazil; ^2^ Mogi das Cruzes University Mogi das Cruzes Brazil; ^3^ Bioengineering & Biomaterials Group, School of Pharmaceutical Sciences São Paulo State University (UNESP) Km 01 Araraquara‐Jaú Road, Araraquara São Paulo, BR Brazil; ^4^ Department of Genetics, Microbiology and Immunology, Institute of Biosciences Universidade Estadual Paulista (UNESP) Botucatu Brazil; ^5^ Department of Biology Maynooth University Maynooth Ireland

**Keywords:** Animal use alternatives, animal disease model, biomedical research, *Galleria mellonella*, translational research

## Abstract

Larvae of the greater wax moth *Galleria mellonella* have become an increasingly important *in vivo* model for biomedical research, providing a practical, ethical, and biologically relevant alternative to vertebrate organisms. Its suitability as a model system lies in its low maintenance cost, ease of handling, and ability to survive at both ambient and mammalian body temperatures. Most importantly, *G. mellonella* exhibits an innate immune system with functional and structural parallels to that of mammals, allowing meaningful insights into infection dynamics and immune responses. This review summarizes the biological and immunological foundations that underpin the use of *G. mellonella* in experimental research and examines its expanding range of applications. The model has been successfully employed to study microbial pathogenicity, antimicrobial efficacy, host–pathogen interactions, and toxicological responses. In recent years, its use has extended to emerging fields such as nanomedicine, immunomodulation, and environmental biotechnology, reflecting its growing translational value. The adoption of *G. mellonella* also aligns with current ethical principles in science, particularly the 3Rs framework (replacement, refinement, and reduction), by minimizing the use of vertebrate animals while maintaining robust experimental outcomes. However, certain challenges persist, including the lack of adaptive immunity and the need for methodological standardization to enhance data reproducibility and comparability across laboratories. Collectively, the growing body of evidence supports *G. mellonella* as a reliable and versatile experimental model that bridges the gap between invertebrate and mammalian systems. Continued methodological refinement and integration with molecular and omics approaches are expected to further consolidate its role in translational and preclinical research. © 2026 The Author(s). *Current Protocols* published by Wiley Periodicals LLC.

## INTRODUCTION

The use of animal models has played a key role in biomedical science, providing essential evidence on the pathophysiology of various diseases and contributing to the development of new treatments. These models allow the evaluation of the pharmacodynamics, pharmacokinetics, and toxicity of experimental compounds, ensuring their safety and efficacy (Gorzalczany & Rodriguez Basso, 2021).

Among the available *in vivo* models, rodents, mainly mice and rats, have been widely used due to their similarity to human metabolism, body temperature, and immune system, making them valuable tools for studying pathogenesis, pharmacology, and toxicology (Ménard et al., 2021). However, the use of these animals raises important ethical issues, as they are sentient organisms and their use entails high costs and specialized infrastructure (Andersen & Winter, 2019).

In response to these concerns, Russell and Burch, in 1959, introduced the principle of the 3Rs (*Reduction, Refinement, Replacement*), which guides the ethical design of animal experiments. These principles aim to reduce the number of animals used, refine procedures to minimize pain and suffering, and promote the development of alternative methods that can replace the use of vertebrates whenever possible (Andersen & Winter, 2019; Gorzalczany & Rodriguez Basso, 2021).

In this context, invertebrate models have emerged as a promising alternative (Freires et al., 2023; Ménard et al., 2021). Organisms such as *Caenorhabditis elegans*, *Drosophila melanogaster*, *Manduca sexta*, and *Galleria mellonella* offer ethical and practical solutions, as they do not require ethics committee approval and have several advantages, including low cost, anatomical simplicity, ease of management, short life cycle, and the possibility of large‐scale experiments.

The nematodes *C. elegans* and the flies *D. melanogaster* were the first invertebrate models widely explored, contributing significantly to the elucidation of biological, genetic, and toxicological processes (Freires et al., 2023). Despite these contributions, both organisms are sensitive to temperatures above 30°C, which limits their applicability to studies involving human pathogens that require 37°C for growth and virulence factor expression (Singulani et al., 2018). In this regard, the larvae of *G. mellonella* have gained prominence for their ability to withstand temperature fluctuations between 25°C and 37°C, thereby recreating a microenvironment conducive to the development of pathogenic microorganisms. In addition, they exhibit remarkable similarities to the innate immune system of mammals, which has driven their use as experimental models in biomedical research (Ménard et al., 2021; Singulani et al., 2018).

Scientific interest in *G. mellonella* has grown exponentially, as reflected in the increasing number of publications addressing everything from basic pathogenesis mechanisms to translational and preclinical trials, including emerging applications involving nanomaterials (Dinh et al., 2021). A recent search in the PubMed database for the term “*Galleria mellonella*” identified approximately 4000 articles published between 1938 and 2025, with more than 80% of them from 2010 onwards. These data demonstrate the consolidation of this insect as a relevant model in several areas of biomedical research. Despite its recognized potential, the lack of methodological standardization and the scarcity of experimental details in some studies still pose challenges to the reproducibility and comparability of results.

In view of this, *G. mellonella* stands out as a versatile, accessible, and biologically relevant model, capable of reproducing complex responses associated with infection and immunity, and of evaluating new therapeutic compounds. Given its broad applicability and growing scientific interest, it is essential to gather and systematize up‐to‐date information on its use, development, standardization, and experimental potential to provide a solid basis for the reproducible use of this model in biomedical studies.

## OVERVIEW AND CONCEPTS

### *Galleria mellonella* as an *in vivo* model: taxonomy, habitat, and structure

*G. mellonella* is a species of moth in the order Lepidoptera, family *Pyralidae*, subfamily *Galleriinae*, phylum Arthropoda, and class Insecta (Marena et al., 2025; Ménard et al., 2021; Serrano et al., 2023). It is a widely distributed moth, known for parasitizing hives of bees of the genus *Apis*, especially *A. mellifera* and *A. cerana*. These insects are found in hives, wasp nests, and also in places where wax is stored (Ménard et al., 2021).

In the wild, the larvae feed on honey, pollen, and wax, which can cause a condition known as galleriasis in hives. This infestation causes significant damage by forming silk‐lined tunnels in the honeycombs, which entangle and obstruct the bees' movement, resulting in population decline and considerable economic losses. Currently, *G. mellonella* has a wide geographical distribution, occurring in 77 countries, particularly in regions with mild to tropical climates. Moreover, its spread is expected to increase with global climate change (Serrano et al., 2023).

**Figure 1:**
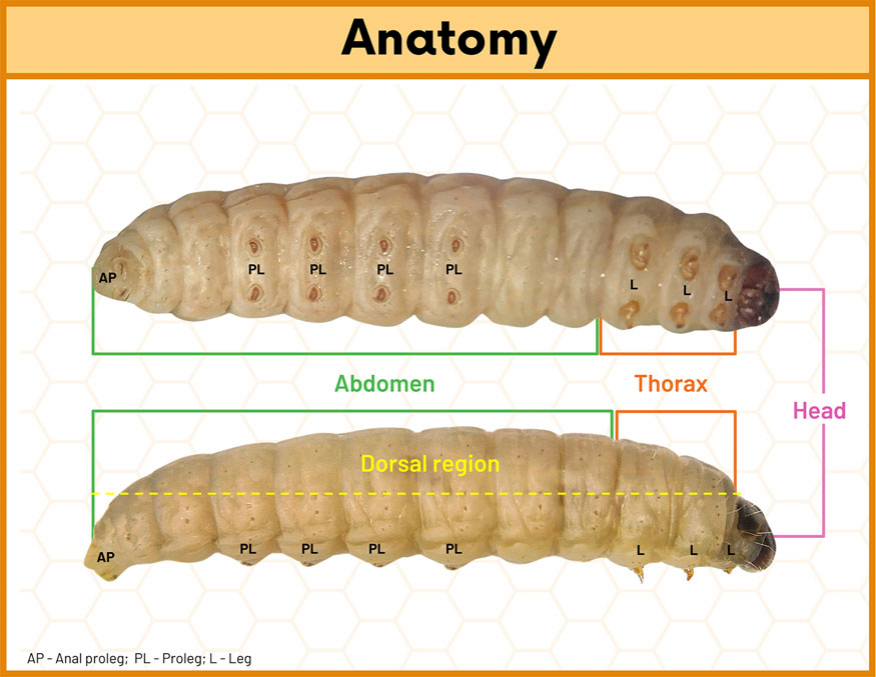
Anatomy of the *Galleria mellonella* larva. The main body regions of the larva of *G. mellonella* are the head, thorax with three pairs of legs (L), and the abdomen with four pairs of prolegs (PL) and one pair of anal prolegs (AP), and the dorsal region.

Anatomically, larvae have four main regions: head, thorax (with three segments and six legs), segmented abdomen (with four pairs of prolegs) (Marena et al., 2025; Ménard et al., 2021) and a specialized dorsal region, described as a “new immune tissue”, where the immune response is predominantly organized (Figure 1) (Serrano et al., 2023). The larval integumentary system is covered by a thick layer, the cuticle, beneath which lies a thin epithelial layer (Ménard et al., 2021). The circulatory system is represented by the hemolymph, which contains immune cells, and the adipose body, which functions as a biosynthetic organ analogous to the mammalian liver (Serrano et al., 2023). The digestive system and silk gland are located within the adipose body, while the ventral region, composed of multiple ganglia, constitutes the nervous system (Ménard et al., 2021).

### Life cycle and experimental use

**Figure 2:**
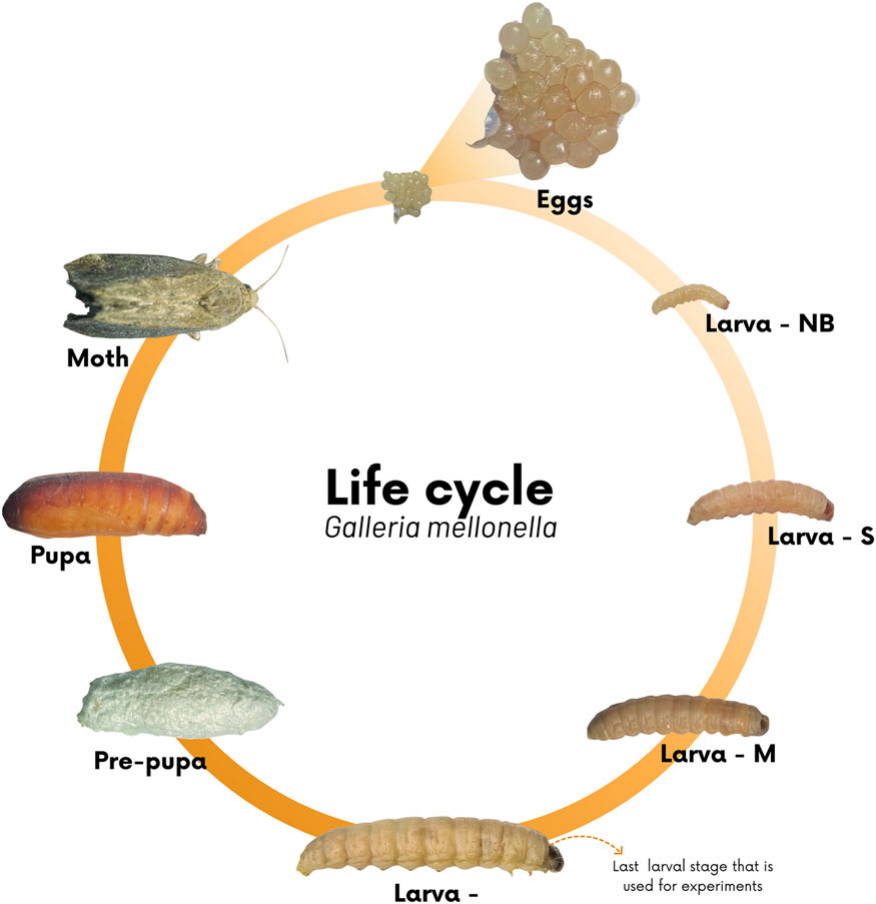
Life cycle of *Galleria mellonella*. The eggs, larvae (or pre‐pupa), pupae, and adults (moth). During the larval stage, progressive growth is observed across the developmental stages: newly hatched (NB), small (S), medium (M), and large (L). During the transition to the pupal stage, the cocoon forms, and then the pupa becomes an adult.

*G. mellonella* is a holometabolous insect, which goes through four stages of development: egg, larva (or pre‐pupa), pupa, and adult, characterizing a complete metamorphic cycle (Figure 2) (Ménard et al., 2021). This cycle is influenced by several environmental factors, such as temperature, diet, humidity, cannibalism, and food competition (Serrano et al., 2023). Under ideal conditions (25°C to 39°C and high humidity), the full cycle lasts six to eight weeks, and can occur four to six generations per year (Marena et al., 2025; Serrano et al., 2023).

The female lays 50 to 150 clustered spherical eggs, which vary in color from white to light pink. Hatching occurs according to ambient temperature: between three and eight days at 24°C to 27°C, and can last for months at lower temperatures (10°C to 16°C) (Ménard et al., 2021; Serrano et al., 2023). The newly hatched larvae grow to 1 to 3 mm in length and pass through 8 to 10 stages. The larval period can range from 28 days to six months, depending on environmental conditions, and is strongly reduced at low temperatures (4°C) (Serrano et al., 2023).

The larvae have a creamy‐white body and a dome‐shaped, reddish‐brown head, which acquires a grayish color as they develop. The tubular body is adapted to process and store food (Serrano et al., 2023). At this stage, they can be classified according to size as small (0.5–10 mm), medium (10–20 mm), or large (>20 mm) (Marena et al., 2025).

In the final larval instar, known as the prepupal stage, the larvae cease feeding, become less mobile, and produce silk threads to build cocoons, in which they will remain until pupation. The pupae, immobilized inside their cocoons, can remain in this stage for up to 9 weeks without feeding before emerging as adult moths (Ménard et al., 2021; Serrano et al., 2023).

Adult moths are light brown to reddish‐brown, nocturnal, sensitive to light, and do not feed, with an average life expectancy of 12 to 21 days. Males and females differ slightly in appearance and longevity; males are smaller, lighter, and tend to live longer (Ménard et al., 2021; Serrano et al., 2023).

### Immune system of *Galleria mellonella*

**Figure 3:**
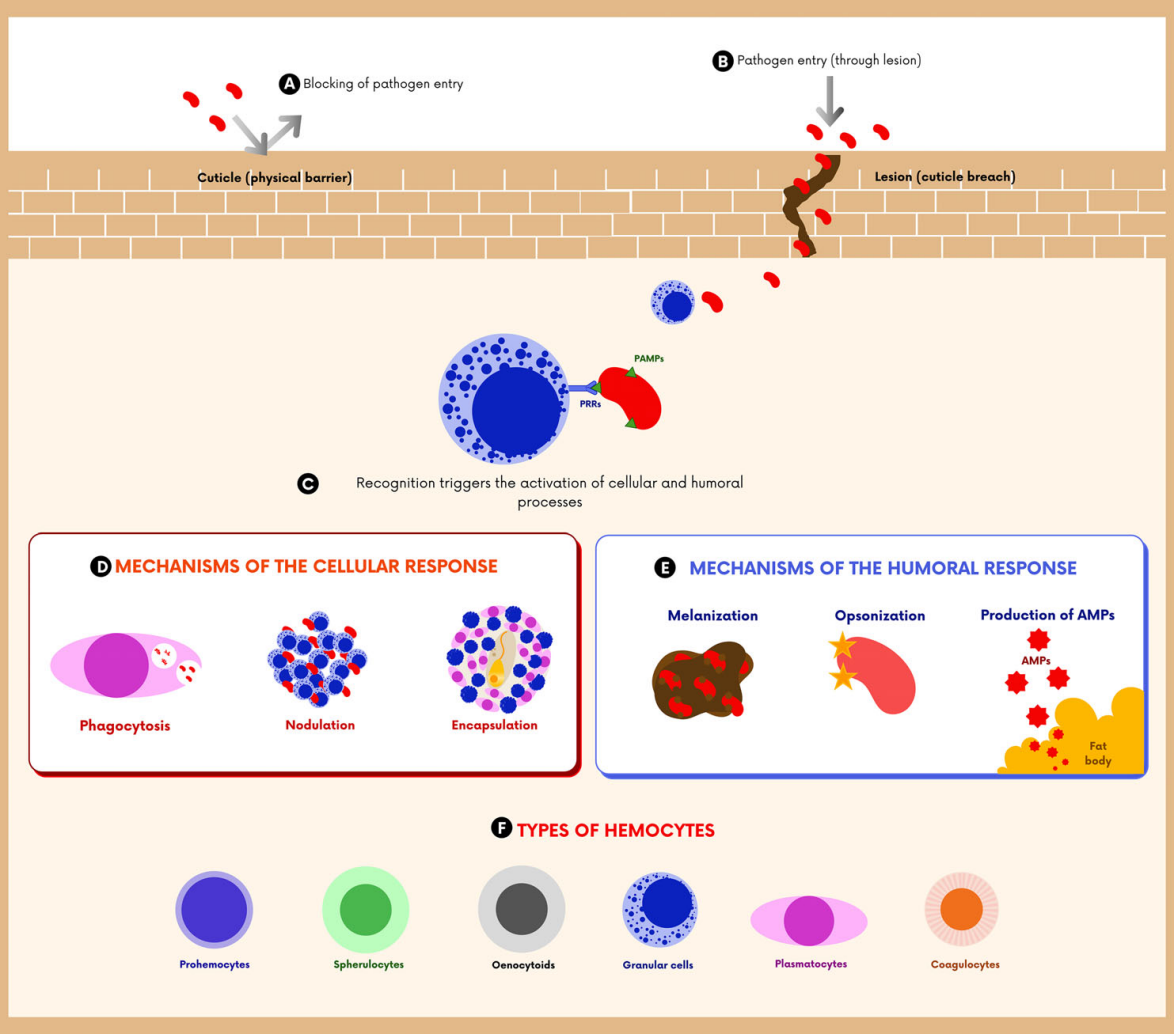
Immune system: A) The cuticle acts as a physical barrier, blocking and preventing the entry of pathogens. B) When this cuticle ruptures, pathogens can enter through the lesion. C) Upon entry, pathogens are recognized by pattern recognition receptors (PRRs) that detect pathogen‐associated molecular patterns (PAMPs), stimulating and activating cellular and humoral immune responses. D) The cellular response is mediated by hemocytes, which are responsible for pathogen recognition, phagocytosis, nodulation, and encapsulation processes. E) The humoral response is responsible for the opsonization of pathogens, the production of antimicrobial peptides (AMPs), and the melanization process. F) Types of hemocytes present in hemolymph: plasmatocytes, granular cells, prohemocytes, coagulocytes, spherulocytes, and enocytoids.

For experimental purposes, the final larval stage of *G. mellonella* is the most widely used because, in this phase, the immune system is fully developed, providing more consistent responses in infection models. This system is composed exclusively of innate immunity mechanisms, which encompass cellular and humoral responses (Figure 3). Although the species lacks adaptive immunity, it has several genes orthologous to vertebrate genes involved in general defense functions, including Toll‐like receptors, components of the NF‐κB signaling cascade, and enzymes associated with oxidative stress responses. This functional similarity makes the model particularly valuable in preclinical studies of host‐pathogen interactions, enabling the investigation of immunologically relevant processes (Marena et al., 2025).

### Cellular immune response

Cellular immunity is mediated by hemocytes (Figure 3F), leukocyte‐like cells present in the hemolymph and bound to internal organs, especially around the digestive tract and adipose body. The number of these cells can vary with the stage of life and the level of stress induced by microorganisms (Ménard et al., 2021).

Pathogen recognition by hemocytes occurs in two ways: through pattern recognition receptors (PRRs) that detect pathogen‐associated molecular patterns (PAMPs), such as lipopolysaccharides, peptidoglycans, lipoteichoic acids, and glucans (Figure 3C), which mediate phagocytosis, encapsulation, and nodulation (Figure 3D); and via indirect recognition through humoral receptors that act as opsonins (Figure 3E) (Marena et al., 2025). Both humoral and cell surface receptors are involved in this recognition, a key process for detecting and engulfing invading pathogens (Serrano et al., 2023).

There are six main types of hemocytes, classified by their microscopic appearance: plasmatocytes (involved in phagocytosis), granular cells (the most abundant), prohemocytes (progenitor cells), coagulocytes (involved in hemolymph coagulation), spherulocytes (related to the secretion of the cuticular component), and oenocytoids, which participate in the melanization pathway and can release extracellular traps to capture pathogens (Figure 3D) (Marena et al., 2025; Ménard et al., 2021).

Nodulation is another essential cellular mechanism, characterized by the rapid formation of nodules or granuloma‐like structures composed of aggregated hemocytes that surround invading pathogens, thus limiting their spread within the hemolymph. Nodulation is typically triggered during systemic infections, especially in response to high microbial loads or the presence of pathogen‐associated molecular patterns. It represents one of the earliest and most quantitatively significant cellular immune reactions in insects. These nodules are frequently encapsulated by melanin, a process linked to the activation of the prophenoloxidase cascade, which contributes to both the immobilization and elimination of the pathogen (Figure 3.D) (Marena et al., 2025).

### Humoral immune response

The humoral response is mediated by soluble molecules in hemolymph that act directly against pathogens or enhance the actions of immune cells. Among the main components are opsonins, antimicrobial peptides (AMPs), melanin, extracellular nucleotides, and protease inhibitors (Figure 3E) (Serrano et al., 2023).

Opsonins, such as apolipophorin‐III (apoLp‐III), peptidoglycan recognition proteins (PGRPs), hemolin, and GmCP8, recognize bacterial components and facilitate phagocytosis (Marena et al., 2025; Serrano et al., 2023). In addition to transporting lipids, apoLp‐III stimulates the production of reactive oxygen species (ROS) and the synthesis of AMPs, such as cecropins (Ménard et al., 2021).

AMPs, including cecropins, moricins, defensins, and gallerimycins, are low‐molecular‐weight peptides that destroy microbial membranes, leading to cell lysis (Ménard et al., 2021). These compounds are mainly produced in adipose tissue, hemocytes, the digestive and reproductive tracts, and salivary glands. To date, more than 20 different types of AMPs have been identified in *G. mellonella* (Ménard et al., 2021; Serrano et al., 2023).

Another fundamental defense mechanism is melanization, catalyzed by phenoloxidase, an enzyme that activates prophenoloxidase. This process results in the deposition of melanin around the microorganisms, promoting encapsulation and coagulation (Serrano et al., 2023). The intensity and speed of this response vary according to virulence and microbial load (Ménard et al., 2021).

In addition, insect metalloproteinase inhibitors (IMPIs) neutralize pathogenic enzymes that degrade immune proteins (Ménard et al., 2021), while lysozymes hydrolyze bacterial peptidoglycans and modulate larval microbiota (Marena et al., 2025; Ménard et al., 2021; Serrano et al., 2023).

### Immune priming

Although *G. mellonella* does not possess adaptive immunity, its larvae exhibit a phenomenon known as immune priming, which is a form of “memory” of innate immunity. After prior exposure to a pathogen, there is an increase in the density of circulating hemocytes and in the production of AMPs in the hemolymph, leading to a faster, more efficient response to reinfection and increasing the chances of survival. Immune priming is evident in many insect species, although its molecular mechanisms have not yet been fully elucidated. The phenomenon has aroused considerable scientific interest, particularly because of the potential to isolate molecules with antimicrobial potential (Sheehan, Farrell, & Kavanagh, 2020).

## HANDLING

*G. mellonella larvae* can be acquired commercially, in several independent breeding sites, since they are widely sold as food for reptiles and birds raised in captivity or as fishing bait (Serrano et al., 2023). However, for scientific purposes, using larvae raised in a laboratory environment is recommended because commercial suppliers maintain highly variable breeding and management conditions, with significant differences in temperature, humidity, and diet (de Jong et al., 2022; Serrano et al., 2023). In addition, transport can expose the larvae to sudden temperature changes, periods of starvation, and physical stress. These factors compromise their health and can alter their metabolism, resulting in inconsistent experimental data (de Jong et al., 2022).

**Figure 4:**
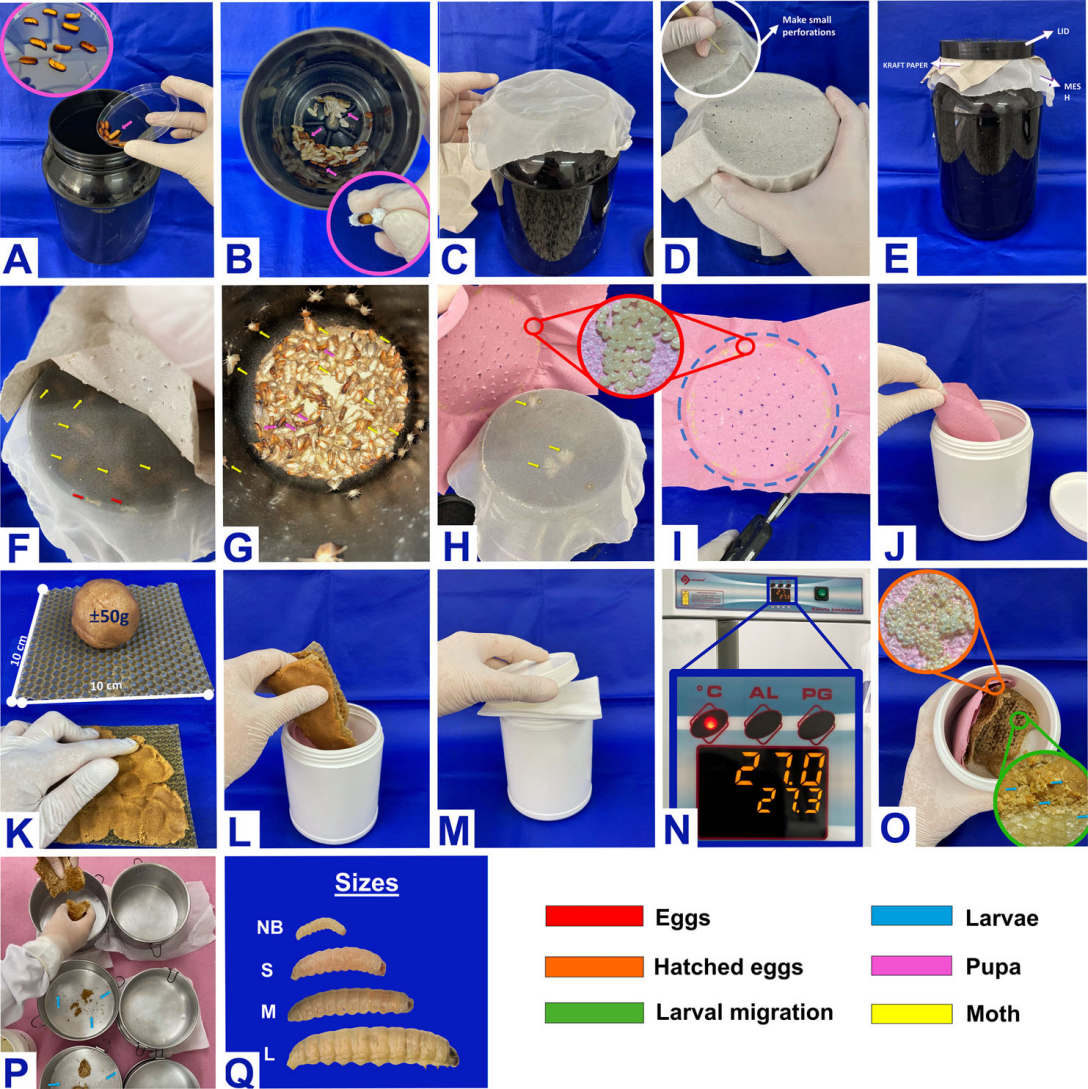
Laboratory breeding of *G. mellonella*. A to E illustrate the care of the pupal stage. (A) The pupae are transferred to the dark pot. (B) The pupae may or may not be encapsulated (highlighted in pink). (C) Placement of a voile to prevent the moths from escaping during egg collection. (D) Cover with paper and make holes for ventilation (highlighted in white) where the eggs will be placed. (E) Finally, the vented lid is placed, leaving the sequence: voile, paper, and lid. From Figure F to G, the care of the moth stage can be observed. (F) Through the voile, it is possible to observe moths (highlighted in yellow) and eggs (highlighted in red). (G) Internal view, presence of pupae (pink) and moths (yellow). H to N illustrate egg care. (H) Egg collection, removed from the paper with the eggs (highlighted in red). (I) Removal of excess paper without eggs. (J) Transfer the paper with eggs to an opaque plastic container. (K) Preparation of the wax (∼10 × ∼10 cm) and distribution of 50 g of feed on one side of the wax. (L) Transfer the feed to the container containing the paper with the eggs. (M) Cover the container with paper towels and close with a vented lid. (N) Incubation at 27°C. O to Q illustrate larval care. (O) Hatched eggs (highlighted in orange) and larval migration, from paper to wax with feed (highlighted in green). (P) Distribution of larvae in aluminum containers (highlighted in blue). (Q) The larvae will be cared for weekly, with cleaning and size divisions into newborn (NB), small (S), medium (M), and large (L).

Controlled breeding in the laboratory, therefore, reduces these variations and brings advantages such as easy management, low maintenance costs, and the possibility of large‐scale reproduction, which favors standardization and experimental reproducibility (Figure 4) (Pereira et al., 2020; Serrano et al., 2023). To achieve these benefits, it is essential to adopt appropriate management practices from the pupal stage through to the collection of larvae destined for the experiments (Jorjão et al., 2018). Pupae should be packed in opaque plastic or glass containers, approximately 30 cm high, protected from light, with perforated lids for ventilation. These containers should be kept at room temperature, without adding food (Figure 4A‐E). Because males can fertilize multiple females, there is no need to strictly control the sex ratio, and females produce many eggs (Jorjão et al., 2018).

To lay the eggs, a filter paper covered by voile fabric must be inserted into the upper part of the container (Figure 4C‐D). After oviposition, the filter paper should be carefully transferred to a new opaque, perforated container (Figure 4H‐J), with a paper towel between the lid and the body of the container to prevent the escape of newly hatched small larvae (Figure 4M). As eggs are extremely sensitive, this transfer must be carried out gently. In this new environment, wax and feed are supplied (Figure 4K), maintaining an average temperature of 27°C to support larval development (Figure 4N). When the larvae reach about 1 cm, it is recommended to clean the container to remove webs, cocoons, and larval feces. It is also recommended to separate them by size (newborn, small, medium, and large), to avoid cannibalism (Figure 4O‐Q). Each group should be transferred to new containers with fresh food, which should be replaced every two or three days (Jorjão et al., 2018).

The diet of larvae destined for research is usually composed of honey, grains, glycerol, dry yeast, beeswax, and powdered milk, that is, low‐cost and easy‐to‐prepare ingredients (Dinh et al., 2021; Jorjão et al., 2018). However, the formulation may vary according to the experimental objectives and the conditions of each laboratory (Dinh et al., 2021). It is important to consider that dietary differences influence the intestinal microbiota of larvae and, consequently, their immune response. Therefore, understanding the impact of nutrition is essential, as inadequate nutrient composition can compromise development, increase susceptibility to infections, or lead to larval mortality (Dinh et al., 2021; Serrano et al., 2023).

## EXAMPLES OF KEY FINDINGS USING THE MODEL

**Figure 5:**
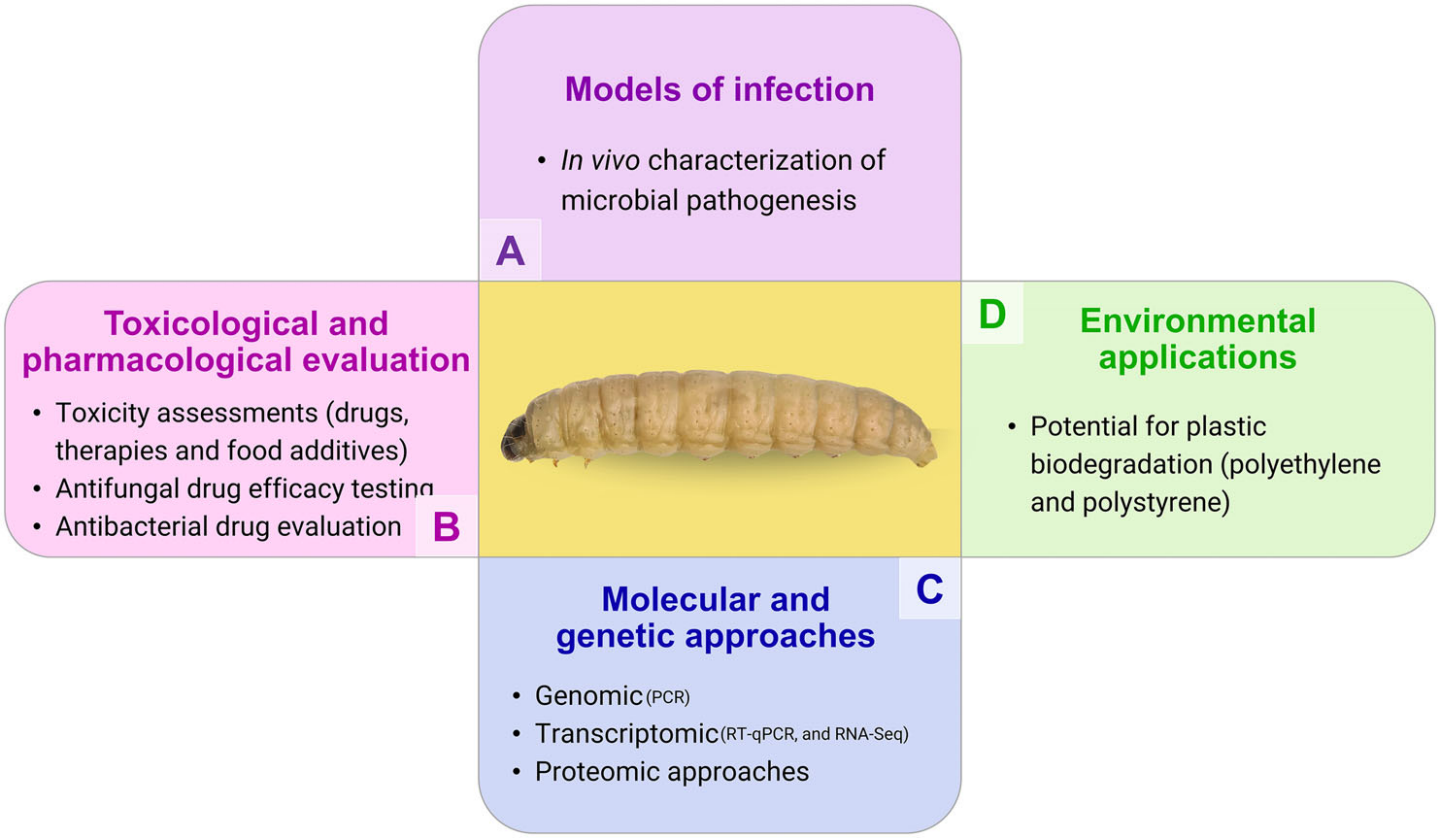
Applications of the use of *G. mellonella* larvae. (A) As models of infection, (B) toxicological and pharmacological evaluation, (C) molecular and genetic approaches, and (D) environmental applications.

As previously mentioned, the versatility of *G. mellonella* has enabled its use across various areas of biomedical research. The model has been successfully employed in a wide range of applications, including *in vivo* characterization of microbial pathogenesis, toxicity assessments, antifungal drug efficacy testing, and antibacterial drug evaluation (Figure 5A‐B) (Piatek, Sheehan, & Kavanagh, 2021). Due to its multifunctionality, it has served as an experimental model, contributing to significant advances in several areas of research. In a pivotal study, Altincicek et al. (2007) demonstrated that bacterial proteases, specifically thermolysin‐like metalloproteinases, can activate serine protease cascades, the prophenoloxidase system, and the melanization response in *G. mellonella*.

These findings are crucial for elucidating how the larval immune system detects endogenous danger signals and recognizes molecular patterns associated with microbes during infection (Figure 5.A). The similarity of these immune recognition mechanisms with those observed in mammals reinforces the relevance of *G. mellonella* as a translational model for the study of host‐pathogen interactions. In another line of research, Mukherjee et al. (2013) employed the *G. mellonella* model to investigate brain infection and the activation of neural repair mechanisms triggered by the human pathogen *Listeria monocytogenes*. Their study revealed important immune responses, including increased melanization, reduced circulating hemocytes, and formation of pigmented lesions in brain tissue. These findings broaden our understanding of the neuroinflammatory effects of *L. monocytogenes infection* and highlight the potential of *G. mellonella* as a model to study central nervous system involvement in microbial pathogenesis. Expanding the scope of applications, Maguire et al. (2016) used *G. mellonella larvae* to evaluate the toxicity of food preservatives administered orally and by intra‐hemocelic injection. The study revealed a strong statistical correlation between the toxicity results observed in *G. mellonella* and those obtained from established mammalian models and cell culture systems. These results support the validity of *G. mellonella* as a reliable alternative model for preliminary toxicological screening of food additives, offering an economically and ethically advantageous approach to toxicity assessment (Figure 5B).

Another notable and emerging application of *G. mellonella* larvae is their potential for plastic biodegradation, a property of particular relevance (Figure 5D). In a global context marked by the overproduction and accumulation of plastic waste, which pose serious environmental and socioeconomic threats, the ability *of G. mellonella* to degrade various types of plastics, such as polyethylene and polystyrene, represents a promising and increasingly investigated biotechnological approach. This unique capability underscores the potential of these larvae as alternative tools to address one of the most pressing environmental challenges of our time (Burd et al., 2025).

The *G. mellonella* larval model has also gained prominence in nanotoxicology research. With the rapid expansion of nanotechnology in industrial and biomedical contexts, the evaluation of the efficacy and safety of polymers, nanocomposites, and nanoparticles *in vivo* has become increasingly important. G*. mellonella* larvae offer a practical and ethically favorable platform for such evaluations, allowing the investigation of toxicological profiles and therapeutic efficacy. This model has proven particularly valuable in infection‐based studies, where nanomaterials are evaluated for their antimicrobial activity under physiologically relevant conditions (Villani et al., 2025). Notably, the G*. mellonella* model is also gaining increasing relevance in molecular‐level research (Figure 5C). The changes resulting from infection, whether in the host larvae or in the pathogen, can be effectively analyzed through genomic, transcriptomic, and proteomic approaches. Large‐scale molecular biology studies, particularly those involving omics technologies and big data, benefit from this model due to its utility for in vivo validation while maintaining lower cost and logistical simplicity compared to mammalian models. Notably, *G. mellonella* is already being used in high‐throughput omics studies of host‐pathogen interactions, offering a scalable and ethically preferable alternative to mammalian models (Dinh et al., 2021).

## TECHNOLOGICAL INNOVATIONS IN INFECTION METHODS AND TECHNIQUES FOR EVALUATING INFECTION PARAMETERS IN LARVAE OF *Galleria mellonella*

Although the *G. mellonella* model is well established, several strategies for inducing infections have been developed to broaden its applicability. Among them, the methods of injection, plaque tracking, and burning stand out. The injection method is the most widely used approach for inoculating larvae with various pathogens, including bacteria and fungi. In this strategy, the microbial inoculum is introduced directly into the hemolymph, usually through the last proleg, using a syringe. This technique simulates a systemic infection similar to that observed in the pathogen's natural host. In addition, because larvae can survive over a wide temperature range, *G. mellonella* can be used to study microorganisms that develop under both environmental (∼25°C) and human physiological (∼37°C) conditions (Carmo et al., 2025).

In addition, an alternative method known as “plate screening” has been described to evaluate the virulence of dermatophytes, pathogenic filamentous fungi, using G. *mellonella larvae*. In this trial, the larvae are incubated directly on previously grown fungal cultures (Li et al., 2010). In addition to this technique, Achterman et al. (2011) tested two other approaches to external inoculation: rolling the larvae on dermatophyte lawns and immersing them in conidia suspension. The development of superficial infections was subsequently compared between the three methods. Although the plate screening technique has shown promising results, these external inoculation strategies have important limitations. In screening, for example, larvae can feed or move between colonies, making it difficult to control the infectious dose and compromising reproducibility. In addition, as the larvae are not sterile, there is a risk of contamination by environmental microorganisms, which prevents conclusive attribution of infections only to the dermatophytes of interest (Li et al., 2010).

Recent advances have also introduced the burn wound model in *G. mellonella*, which offers relevant information on healing and infection dynamics. Notably, this invertebrate undergoes re‐epithelialization during tissue repair, reproducing aspects of the healing process observed in humans. The model successfully reproduces core elements of trauma and burn infection typical of mammalian systems, such as the correlation between lesion size and survival prognosis, the benefits of rehydration, and the increased mortality associated with topical infection (Piatek et al., 2021). In addition, it has been shown to be effective in treating superficial infections caused by bacterial and fungal pathogens, with evidence of microbial dissemination in the hemolymph (Maslova et al., 2020; Terra Garcia et al., 2025).

Once infection is established, various parameters can be evaluated to assess host response, infection progression, and treatment efficacy. Among these, larval survival is one of the most commonly used, usually analyzed with Kaplan‐Meier curves, which track larval viability for 5 to 10 days. Larval death may reflect microbial virulence, the toxicity of the tested compound, or insufficient treatment efficacy in the experimental model (Marena et al., 2025).

**Figure 6:**
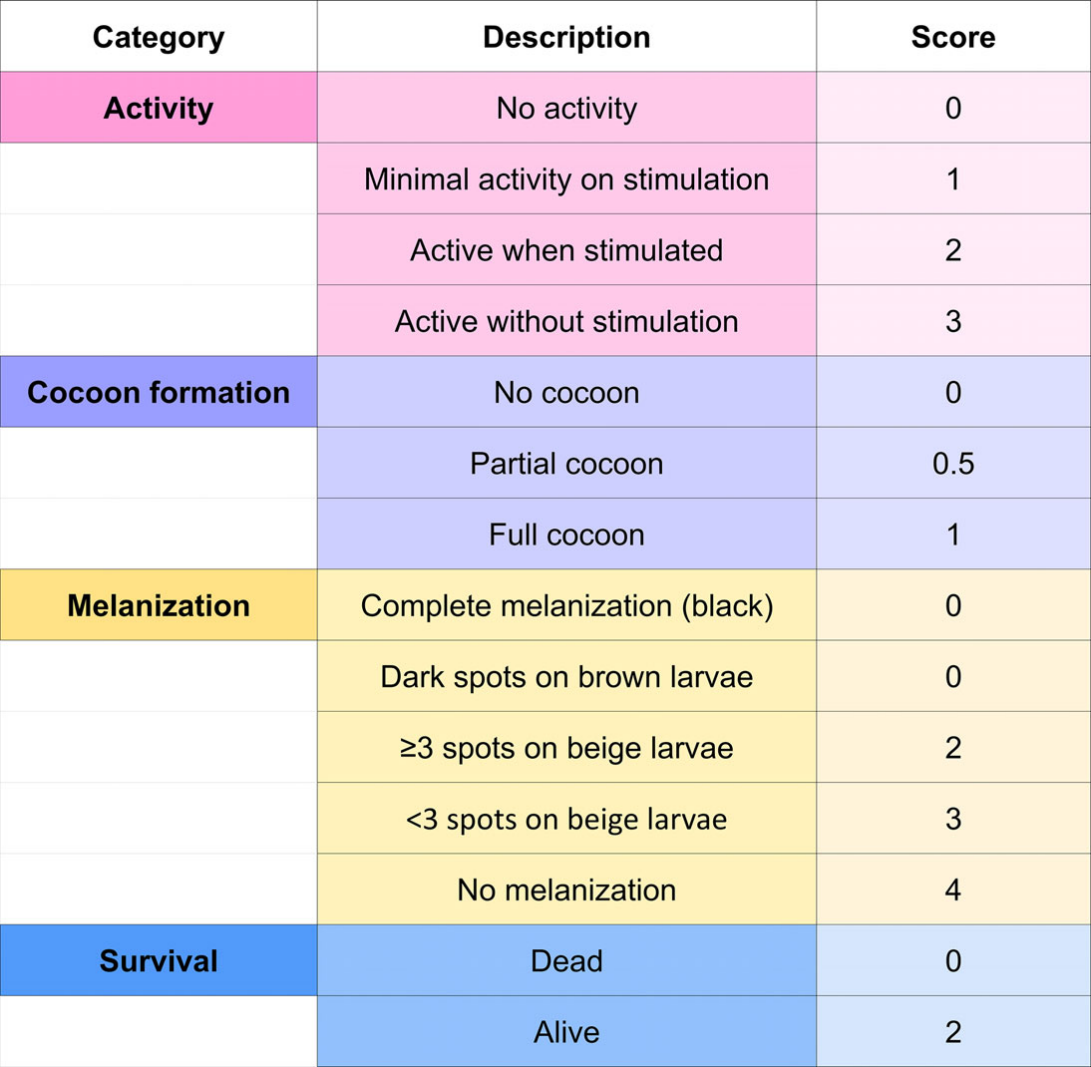
Score table for evaluating the health of *G. mellonella* larvae.

**Figure 7:**
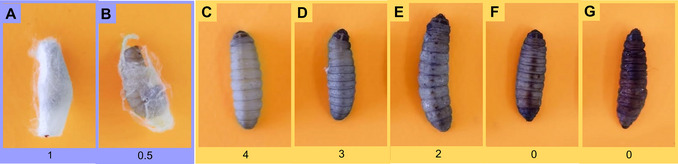
Visual representation of the health of *Galleria mellonella* larvae. Formation of a full cocoon (A) and a partial (B). Melanization levels: (C) non‐melanization, (D and E) beige larva, (F) brown larva, and (G) complete melanization (black).

Progression of infection is often assessed through the larval health index and quantification of microbial load. The health index is based on four main criteria: survival, activity, cocoon formation, and melanization. High levels of activity and cocoon formation indicate better larval condition, while melanization (innate immune response associated with melanin production), when exacerbated, reflects greater severity of infection (Figures 6 and 7) (Loh et al., 2013).

In addition to evaluating the health index, quantifying microorganisms in the hemolymph and counting circulating hemocytes are essential parameters for monitoring infectious dynamics. Microbial load can be measured by classical methods, such as colony‐forming unit (CFU) counting, or by advanced imaging techniques, molecular and proteomic analyses. While CFU counting allows direct comparisons between experimental groups, imaging approaches, including bioluminescence imaging, confocal microscopy, and marker‐free multimodal techniques, provide real‐time information on the distribution and spread of pathogens (Quansah et al., 2022).

Molecular analyses (PCR, RT‐qPCR, and RNA‐Seq) complement these evaluations by enabling detection of nucleic acids and analysis of gene expression profiles. When combined with proteomic approaches, which identify changes in protein abundance in specific contexts, these techniques provide an integrated view of the molecular changes induced by infection. This integration facilitates the identification of microbial virulence factors and the understanding of host immune responses (Dinh et al., 2021).

The larval model has also proven useful for investigating host responses to genetically modified strains, allowing specific mutations to be correlated with immunological alterations. This capacity, combined with its scalability and the potential to generate functional data at the genomic level, reinforces the value of *G. mellonella* as an alternative model to murine systems, especially for species that are difficult to manipulate (Bruchmann et al., 2021).

Finally, quantifying hemocyte density provides crucial information about larval immune mechanisms. This analysis can be performed using a hemocytometer under light microscopy or more sophisticated methods, such as confocal microscopy and flow cytometry. For example, de Barros et al. (2021) used flow cytometry to distinguish at least five hemocyte subpopulations in *G. mellonella* based on differences in size and granularity. This approach allowed the monitoring of variations in cell composition, offering new insights into immune modulation in the face of infection.

## LIMITATIONS

Despite its benefits, the *G. mellonella* larval model has important limitations that should be considered in experimental design and the interpretation of results. The absence of an adaptive immune system is one of them. As already mentioned, larvae of this insect have only innate immunity; they do not produce antibodies or T and B cells, nor do they develop immunological memory as vertebrates do. This means that studies that rely on adaptive responses, such as vaccines or specific immunotherapy, cannot be performed completely in this model (Bugyna, Kendra, & Bujdáková, 2023).

In addition, there are substantial physiological and anatomical differences in relation to mammals. For example, the larva's metabolism, body structure, open circulatory system (hemolymph rather than blood in closed vessels), lower complexity of certain organs, or the functional absence of some mammalian equivalents limit how much toxicity, pharmacokinetics, and drug dynamics data can be extrapolated. These differences can alter rates of absorption, metabolism, or tissue response to the pathogen or therapeutic agent. Transcriptomic studies show that, although there is a repertoire of conserved immune genes, many molecular aspects remain poorly characterized in the model (Vogel et al., 2011).

Another limitation lies in genetic and molecular constraints: although there are transcriptome data (e.g., at different stages of development or after immune challenge) that help identify immune response genes and conserved pathways, there is a gap in standardized genetic tools, few well‐characterized mutant lines are available, gene banks are still incipient, and not all defense genes/proteins are identified or functionally validated. This makes it difficult, for example, to study deletion or differential expression with a guarantee of comparability and reproducibility between laboratories (Vogel et al., 2011).

Related to this, difficulties arise in standardizing the dose and route of infection. Variability in larval weight, inoculum volume and concentration, injection site, and interval between infection and evaluation can yield significant differences in results. In addition, as already mentioned, there is a lack of standardization between laboratories regarding several critical parameters, such as larval diet, maintenance conditions (temperature and humidity), origin or supplier, larval stage used, and criteria for health evaluation (degree of melanization, mobility, cocoon formation, hemocyte count, etc.). This methodological heterogeneity makes it difficult to replicate the experiments and compare different studies.

Variations in stock, weight, and larval management were observed across studies involving infection and antimicrobial screening, reinforcing the need for uniform, reproducible protocols to ensure greater reliability of the model. A relevant example is the study by Carmo et al. (2025), which evaluated various incubation and egg activation methods under varying temperature conditions. The authors demonstrated that low temperatures (−4°C) prevent eggs from hatching, while abrupt temperature variations can alter larval susceptibility to pathogens and hemocyte recruitment. On the other hand, gradual and controlled changes in temperature are a promising approach. Although they slow the development cycle, they preserve susceptibility and cellular immune response at levels comparable to those observed in the traditional group, which was maintained under stable laboratory conditions for more than a decade.

## CONCLUDING REMARKS

In view of the above, the *G. mellonella* larval model has established itself as a versatile, ethical, and low‐cost experimental tool for investigating infections and evaluating antimicrobial substances. Advances in infection methods and analytical techniques have broadened their applicability across different research niches. However, limitations such as the absence of adaptive immunity, physiological differences from those of mammals, and a lack of standardization between laboratories still affect their reproducibility. Thus, strengthening the model depends on adopting uniform protocols and using integrated approaches to improve the reliability and comparability of results.

### Author Contributions

**Fabiana Alves de Souza Silva**: Conceptualization; data curation; formal analysis; investigation; methodology; writing—original draft. **Maria Eduarda da Silva Costa**: Conceptualization; data curation; investigation; methodology; writing—original draft. **Patrícia Michelle Nagai de Lima**: Investigation; methodology; writing—original draft. **Jaqueline Lemes Ribeiro**: Investigation; methodology; writing—original draft; writing—review and editing. **Betina Sayeg Burd**: Investigation; methodology; writing—original draft. **Rondinelli Donizetti Herculano**: Investigation; methodology; writing—original draft. **Laura Beatriz Borim da Silva**: Research; methodology; writing—original draft. **Paulo Henrique Fonseca Carmo**: Conceptualization; data curation; writing—review and editing. **Luis Augusto de Almeida‐Silva**: Research; methodology; writing—original draft; writing—review and editing. **Kevin Kavanagh**: Conceptualization; project administration; writing—review and editing. **Juliana Campos Junqueira**: Conceptualization; project administration; writing—review and editing. **Maíra Terra Garcia**: Conceptualization; data curation; formal analysis; funding acquisition; research; methodology; project administration; supervision; validation; visualization; writing—original draft; writing—review and editing.

### Conflict of Interest

The authors declare no conflict of interest.

## Data Availability

Data sharing not applicable to this article as no datasets were generated or analysed during the current study.

## References

[cpz170361-bib-0001] Achterman, R. R. , Smith, A. R. , Oliver, B. G. , & White, T. C. (2011). Sequenced dermatophyte strains: Growth rate, conidiation, drug susceptibilities, and virulence in an invertebrate model. Fungal Genetics and Biology, 48(3), 335–341. 10.1016/j.fgb.2010.11.010 21145410 PMC3035951

[cpz170361-bib-0002] Altincicek, B. , Linder, M. , Linder, D. , Preissner, K. T. , & Vilcinskas, A. (2007). Microbial Metalloproteinases Mediate Sensing of Invading Pathogens and Activate Innate Immune Responses in the Lepidopteran Model Host *Galleria mellonella* . Infection and Immunity, 75(1), 175–183. 10.1128/iai.01385-06 17074843 PMC1828416

[cpz170361-bib-0003] Andersen, M. L. , & Winter, L. M. F. (2019). Animal models in biological and biomedical research—Experimental and ethical concerns. Anais Da Academia Brasileira de Ciências, 91, e20170238. 10.1590/0001-3765201720170238 28876358

[cpz170361-bib-0004] Bruchmann, S. , Feltwell, T. , Parkhill, J. , & Short, F. L. (2021). Identifying virulence determinants of multidrug‐resistant Klebsiella pneumoniae in *Galleria mellonella* . Pathogens and Disease, 79(3), ftab009. 10.1093/femspd/ftab009 33512418 PMC7981267

[cpz170361-bib-0005] Bugyna, L. , Kendra, S. , & Bujdáková, H. (2023). *Galleria mellonella*—A Model for the Study of aPDT—Prospects and Drawbacks. Microorganisms, 11(6), 1455. 10.3390/microorganisms11061455 37374956 PMC10301295

[cpz170361-bib-0006] Burd, B. S. , Mussagy, C. U. , Bebber, C. , Sant'Ana Pegorin Brasil, G. , dos Santos, L. S. , Guerra, N. B. , Persinoti, G. F. , Jucaud, V. , Goldbeck, R. , & Herculano, R. D. (2025). Can the insects *Galleria mellonella* and *Tenebrio molitor* be the future of plastic biodegradation? Science of The Total Environment, 969, 178879. 10.1016/j.scitotenv.2025.178879 40022971

[cpz170361-bib-0007] Carmo, P. H. F. , de Lima, P. M. N. , Silva, F. A. , Ribeiro, J. L. , Junqueira, J. C. , & Garcia, M. T. (2025). Impact of egg incubation on hemocyte recruitment and susceptibility of *Galleria mellonella* larvae to pathogens. Frontiers in Microbiology, 16, 1611104. 10.3389/fmicb.2025.1611104 40529588 PMC12170569

[cpz170361-bib-0008] de Barros, P. P. , Rossoni, R. D. , Garcia, M. T. , Kaminski, V. , de, L. , Loures, F. V. , Fuchs, B. B. , Mylonakis, E. , & Junqueira, J. C. (2021). The Anti‐Biofilm Efficacy of Caffeic Acid Phenethyl Ester (CAPE) In Vitro and a Murine Model of Oral Candidiasis. Frontiers in Cellular and Infection Microbiology, 11, 700305. 10.3389/fcimb.2021.700305 34408988 PMC8366685

[cpz170361-bib-0009] de Jong, A. W. , van Veldhuizen, D. , Groot, A. T. , & Hagen, F. (2022). Standardized methods to rear high‐quality *Galleria mellonella* larvae for the study of fungal pathogens. Entomologia Experimentalis et Applicata, 170(12), 1073–1080. 10.1111/eea.13237

[cpz170361-bib-0010] Dinh, H. , Semenec, L. , Kumar, S. S. , Short, F. L. , & Cain, A. K. (2021). Microbiology's next top model: *Galleria* in the molecular age. Pathogens and Disease, 79(2), ftab006. 10.1093/femspd/ftab006 33476383

[cpz170361-bib-0011] Freires, I. A. , Morelo, D. F. C. , Soares, L. F. F. , Costa, I. S. , de Araújo, L. P. , Breseghello, I. , Abdalla, H. B. , Lazarini, J. G. , Rosalen, P. L. , Pigossi, S. C. , & Franchin, M. (2023). Progress and promise of alternative animal and non‐animal methods in biomedical research. Archives of Toxicology, 97(9), 2329–2342. 10.1007/s00204-023-03532-1 37394624

[cpz170361-bib-0012] Gorzalczany, S. B. , & Rodriguez Basso, A. G. (2021). Strategies to apply 3Rs in preclinical testing. Pharmacology Research & Perspectives, 9(5), e00863. 10.1002/prp2.863 34609088 PMC8491455

[cpz170361-bib-0013] Jorjão, A. L. , Oliveira, L. D. , Scorzoni, L. , Figueiredo‐Godoi, L. M. A. , Cristina, A. , Prata, M. , Jorge, A. O. C. , & Junqueira, J. C. (2018). From moths to caterpillars: Ideal conditions for *Galleria mellonella* rearing for *in vivo* microbiological studies. Virulence, 9(1), 383–389. 10.1080/21505594.2017.1397871 29130369 PMC5955185

[cpz170361-bib-0014] Li, W. , Metin, B. , White, T. C. , & Heitman, J. (2010). Organization and Evolutionary Trajectory of the Mating Type (MAT) Locus in Dermatophyte and Dimorphic Fungal Pathogens. Eukaryotic Cell, 9(1), 46–58. 10.1128/ec.00259-09 19880755 PMC2805302

[cpz170361-bib-0015] Loh, J. M. S. , Adenwalla, N. , Wiles, S. , & Proft, T. (2013). *Galleria mellonella* larvae as an infection model for group A streptococcus. Virulence, 4(5), 419–428. 10.4161/viru.24930 23652836 PMC3714134

[cpz170361-bib-0016] Maguire, R. , Duggan, O. , & Kavanagh, K. (2016). Evaluation of *Galleria mellonella* larvae as an *in vivo* model for assessing the relative toxicity of food preservative agents | Cell Biology and Toxicology. Cell Biology and Toxicology, 32(3), 209–216. 10.1007/s10565-016-9329-x 27122324

[cpz170361-bib-0017] Marena, G. D. , Thomaz, L. , Nosanchuk, J. D. , & Taborda, C. P. (2025). *Galleria mellonella* as an Invertebrate Model for Studying Fungal Infections. Journal of Fungi, 11(2), 157. 10.3390/jof11020157 39997451 PMC11856299

[cpz170361-bib-0018] Maslova, E. , Shi, Y. , Sjöberg, F. , Azevedo, H. S. , Wareham, D. W. , & McCarthy, R. R. (2020). An Invertebrate Burn Wound Model That Recapitulates the Hallmarks of Burn Trauma and Infection Seen in Mammalian Models. Frontiers in Microbiology, 11, 998. 10.3389/fmicb.2020.00998 32582051 PMC7283582

[cpz170361-bib-0019] Ménard, G. , Rouillon, A. , Cattoir, V. , & Donnio, P.‐Y. (2021). *Galleria mellonella* as a Suitable Model of Bacterial Infection: Past, Present and Future. Frontiers in Cellular and Infection Microbiology, 11, 782733. 10.3389/fcimb.2021.782733 35004350 PMC8727906

[cpz170361-bib-0020] Mukherjee, K. , Hain, T. , Fischer, R. , Chakraborty, T. , & Vilcinskas, A. (2013). Brain infection and activation of neuronal repair mechanisms by the human pathogen Listeria monocytogenes in the lepidopteran model host Galleria mellonella. Virulence, 4(4), 324–332. 10.4161/viru.23629 23348912 PMC3710335

[cpz170361-bib-0021] Pereira, M. F. , Rossi, C. C. , da Silva, G. C. , Rosa, J. N. , & Bazzolli, D. M. S. (2020). *Galleria mellonella* as an infection model: An in‐depth look at why it works and practical considerations for successful application. Pathogens and Disease, 78(8), ftaa056. 10.1093/femspd/ftaa056 32960263

[cpz170361-bib-0022] Piatek, M. , Sheehan, G. , & Kavanagh, K. (2021). *Galleria mellonella*: The Versatile Host for Drug Discovery, In Vivo Toxicity Testing and Characterising Host‐Pathogen Interactions. Antibiotics, 10(12), 1545. 10.3390/antibiotics10121545 34943757 PMC8698334

[cpz170361-bib-0023] Quansah, E. , Ramoji, A. , Thieme, L. , Mirza, K. , Goering, B. , Makarewicz, O. , Heutelbeck, A. , Meyer‐Zedler, T. , Pletz, M. W. , Schmitt, M. , & Popp, J. (2022). Label‐free multimodal imaging of infected *Galleria mellonella* larvae. Scientific Reports, 12(1), 20416. 10.1038/s41598-022-24846-7 36437287 PMC9701796

[cpz170361-bib-0024] Serrano, I. , Verdial, C. , Tavares, L. , & Oliveira, M. (2023). The Virtuous *Galleria mellonella* Model for Scientific Experimentation. Antibiotics, 12(3), 505. 10.3390/antibiotics12030505 36978373 PMC10044286

[cpz170361-bib-0025] Sheehan, G. , Farrell, G. , & Kavanagh, K. (2020). Immune priming: The secret weapon of the insect world. Virulence, 11(1), 238–246. 10.1080/21505594.2020.1731137 32079502 PMC7051127

[cpz170361-bib-0026] Singulani, J. L. , Scorzoni, L. , de Oliveira, H. C. , Marcos, C. M. , Assato, P. A. , Fusco‐Almeida, A. M. , & Mendes‐Giannini, M. J. S. (2018). Applications of Invertebrate Animal Models to Dimorphic Fungal Infections. Journal of Fungi, 4(4), 118. 10.3390/jof4040118 30347646 PMC6308930

[cpz170361-bib-0027] Terra Garcia, M. , Castro Pedroso, L. L. , Do Carmo, P. H. F. , da Silva, L. A. N. , Bueno, T. L. , dos Santos, V. G. R. , Fraga, A. S. , Nagai de Lima, P. M. , Soares da Silva, N. , de Paula, L. R. , de Oliveira, L. D. , Iglesias, B. A. , & Junqueira, J. C. (2025). Functionalization of cationic porphyrins with peripheral platinum(II) complexes to optimize photodynamic therapy against *Candida*‐associated infections: A focus on denture stomatitis and burn wounds. Mycology, 1–20. 10.1080/21501203.2025.2468756 41334526 PMC12667294

[cpz170361-bib-0028] Villani, S. , Calcagnile, M. , Demitri, C. , & Alifano, P. (2025). *Galleria mellonella* (Greater Wax Moth) as a Reliable Animal Model to Study the Efficacy of Nanomaterials in Fighting Pathogens. Nanomaterials, 15(1), 67. 10.3390/nano15010067 39791825 PMC11723170

[cpz170361-bib-0029] Vogel, H. , Altincicek, B. , Glöckner, G. , & Vilcinskas, A. (2011). A comprehensive transcriptome and immune‐gene repertoire of the lepidopteran model host *Galleria mellonella* . BMC Genomics, 12(1), 308. 10.1186/1471-2164-12-308 21663692 PMC3224240

